# Supplementary material for: Patterns of Intron Gain and Loss in Fungi
Source: PLoS Biol. 2004 Nov 30;2(12):e422. doi: 10.1371/journal.pbio.0020422 (PMC532390; doi:10.1371/journal.pbio.0020422)
Supplement: Table S1 — Also available at http://genes.mit.edu/NielsenEtAl/. (4.3 MB ZIP). [file pbio.0020422.st001.zip › NielsenEtAl/html/336.html]

AN7952.1.NCU00796.1.MG00722.1.FG06997.1


```
 CLUSTAL W (1.82) Multiple Sequence Alignments - Introns Inserted


Sequence 1: NCU00796.1	1060 aa
Sequence 2: FG06997.1	997 aa
Sequence 3: AN7952.1	994 aa
Sequence 4: MG00722.1	988 aa
Alignment Length: 1093 aa
Number Identitical Residues: 213 aa
Alignment Score (without introns) 13636


MG00722.1 	MDVVAWVS2CYGHIGAGSVG~IFGDWSRCRR2-------YSVVIPSPGTNTDSFLLDLSK
NCU00796.1	MAPQPSPR~RLR-IGVDVGG~TNTDGVILDP~SLASSSPSKGILAHVKTATTPNPSEGIM
FG06997.1 	MSATTAAR~RLR-VGIDVGG~TNTDGVLIDP~-LKTSGPDRGIIAWHKEPTTTNPSVGIN
AN7952.1  	MGSLP-DH~RYR-IGVDVGG1TNTDAVLIAP~------DSMTIIASHKAPTTPDVTTGIT
          	*   .        :* .  *    *                 ::.     * .       

MG00722.1 	YGSDAVLAS-----HKAVTS----SNVALGVQETLQALLAGAAAAPASDASGR2LAQ---
NCU00796.1	TAITSMFTQLQATHAADIRPEDAIASVTIGTTHFVNAVIERDPSRLSRVAVLR~LSG---
FG06997.1 	NALTKMLS------SASISP-DEVASVTIGTTHFVNAVVERDATRLSRVAVIR~LCG---
AN7952.1  	NAVQTVIE------TASVSL-SSIGCVIVGTTHFVNAVVQRSP-ALRRVAVIR~LCGGPD
          	 .   ::          :   .  . * :*. . ::*::   .      *  * *. ...

MG00722.1 	-AAGKHHP----WLLRHHS-----------RGCNIDGTLIAHLDGDEVRRQATAIRAAG-
NCU00796.1	-PFSKHVPPCVDWPDDMRSLMLG-YHARVKGGLEVDGSLISDIDESEIVEQCKEIRKRGG
FG06997.1 	-PFSKHILPCVDWPSDMKELILG-HHALVKGGLEVDGRLISDINQDEIKAQCAIIKEKG-
AN7952.1  	EGFGRGIPPFTDFPLDLRSCIESPRQYFCHGGYQISGEEISAIDEDEIRRIAAELTADG-
          	.  .:   .  .:    :.   ..       * ::.*  *: :: .*:   .  :   * 

MG00722.1 	IKNVVVVGIGSPMDERHHQEKRARDIIIDELGDET---NVVCSADIAGSGLLARENASIL
NCU00796.1	IKAVVINGVFSPIDTVYRQEERAAEIVRRELGGQE-EVDVVCSKEVANLGFLERENAAVL
FG06997.1 	IKSVVVIGVFSPIDTVERQEERAADIIKGEITG----CDVVCSKEVANLGFLERENAAIL
AN7952.1  	VQNIVISGMYAPLN--NAQEVAVRDILLQTMTSANSKPRITLSHEISGLGFLSRENAAIL
          	:: :*: *: :*::    **  . :*:   : .  :.  :. * :::. *:* ****::*

MG00722.1 	NAAILSFARRTIRAFIGAMARVGLS--CPLYLTSNAGHLLPFSEAMTAPIRIFSSGATNS
NCU00796.1	NASILRFARRTIREFRQAVGRLGLKG-VPVFITQNDGTILKGEKAAELPIKTFSSGPTNS
FG06997.1 	NASILPFARKTIRSFHEPVKRLGLN--CPVFITQNDGTVLSGELAARLPIRTFSSGPTNS
AN7952.1  	NATLRPLAEKTIYAFKKAMRDIFQSNPYTLYLTQNDGSVLSAGEAVDKPIRTFNSGPTNS
          	**::  :*.:**  *  .:  :  ... .:::*.* * :*    *   **: *.**.***

MG00722.1 	IRGAAFLAGTEAIG--------RTGSVVVDIGGTTTDVGYLLGNGYPRLTKSYSDLAGVK
NCU00796.1	MRGAAFLVQGQEKDGKKEGQEQGKAMMVVDVGGTTTDVGLLLPNGFPRQQAAYSELAGVR
FG06997.1 	MRGAAFLVQN----------ELDEAMMVVDIGGTTSDVGILLENGFPRQQAAYSDLSAVR
AN7952.1  	IRGGEFLWRAAGKASGLGQEDRTEPLVVIDIGGTTSDSGLLLPNGLPQMSSVTGLVGGVR
          	:**. **       .    ..     :*:*:****:* * ** ** *:     . :..*:

MG00722.1 	V~NLEMASVESVGLGGGSIVRVDQAPVSVGPQSVGHDLMTRALCFGGDQTTATDIAVAG-
NCU00796.1	M0NFSCPDIKSIGLGGGSIVRKGKNGITIGPDSVGYRINKEALLFGGSTLTTTDCTVLT-
FG06997.1 	M~NFSCPDIKSIGLGGGSIVRR-DNGISVGPDSVGYKLTQEAVVFGGKTLTATDCTVLA-
AN7952.1  	T~NFALPAVESIGLGGGSIIRETDGELTVGPDSVALELLEKAKLFGGDYLTSTDIVAAAG
          	  *:  . ::*:*******:*  .  :::**:**.  :  .*  ***.  *:** ..  .

MG00722.1 	--------GARIEAKGLRGVNLSQAVVASAQARIKKMIETTIDRAKSSPDPCTVILVGGG
NCU00796.1	-----NPEITSIGDASLVKAALTEEETQQFKAIVKSKIEKIIDNMKTSPEDLPAILVGGG
FG06997.1 	-----NPGL-EIGNATLLKDAIYEDELDKVRLAIKHKLEKVIDTMKTSPQDIPVILVGGG
AN7952.1  	IHSPCEPNPFRGMGDTSRLADITADMVSRVREKMRQMIAALVDRTKTQKGDIDVLIVGGG
          	  :....              :        :  ::  :   :*  *:.     .::****

MG00722.1 	SILCP--RELRGVRKVLVPPHAAVANAIGAATANVHGAAETIIHG--ADVATGIAAVKKK
NCU00796.1	AVIAP--DELQGASKVLKPRWSEVANAIGAAIARVSAVVDTIKSTEAKTEKQFLEEIKKD
FG06997.1 	AVIAP--DKLKGASKVLKPQWSQVANAIGAAMARVSAVVDTVKSTVKKSANELLEEVSQE
AN7952.1  	AALIKTDEPLTGVRSLRTVSGAEVANAVGAAISRVSGVIDTVVDTSNQSVKSAQEFVSRS
          	: :  :.  * *. .:     : ****:*** :.* .. :*:              :.:.

MG00722.1 	AVGNAVAKGGDARGPVTVLHEEVAGVP~YVEGQTVIKIEVALPAGHARVYAEMLRTPSEN
NCU00796.1	AVERTIAAG-ASPESVEVVEVEMLPLQ0YVENKTRFVVRAAGDFDFTRSYTSSDDNGKAS
FG06997.1 	AIEKTVKAG-ALRSTVTVVEKEVLPLQ0YIANKTRFVVRATGDFDFSKESVVPETN--EE
AN7952.1  	AVEKAVANG-AKPETVQIAEVTMLPIQ~YVDAKARIVVRAVGELAVVSQGVEEIFG---Q
          	*: .::  *     .* : .  :  :  *:  :: : :...         .        .

MG00722.1 	YEKAVDEEVSGSAGARTGNTGDARQVDAEAPVVNPDCYRPEITSDGHWKVSQTDIHFLSI
NCU00796.1	SEEEAVGKDL----FEPQTVTDTKPKAATSSQVNVLTYTPNVLNR-IWHISETDLTFIST
FG06997.1 	SEEHQEMDSY----EKSAKDQSSKRDTKEEEDFDILAYRPDVRER-TWYISERDVSWIAT
AN7952.1  	CEKHEEAE--------KEEVARSIPAKAADEVDDIQSYRPLIKNR-QWIISTTDLGFIAQ
          	 *:    .              :          :   * * : .   * :*  *: ::: 

MG00722.1 	GCYILGCGGGGSTYTHHLELRQLLH-EGHDVRIVETTSLGDDDVLVPVAGVGTPAVGIER
NCU00796.1	GCYILGTGGGGSPYASMILLRQLLR-SGASVRVVSPADVPDSAAVGCGCGAGSPTVSIEK
FG06997.1 	GCYILGTGGGGSPYGLMIRLRSQLR-NGAIVRVVNPEDLSDNARVGCGGGAGSPTVAIEK
AN7952.1  	GCKVLGSGGGGDPYQEFLKVSALVRKNPGTVRVVSPDYLPDDALVGWTGNMGSPEVSMER
          	** :** ****..*   : :   ::..   **:*..  : *.  :    . *:* *.:*:

MG00722.1 	PGGDGVYHAMQEMEKLMGGH---------FSRLLATEIGGANGVGTLLWGSSKYYSIPTV
NCU00796.1	LQGDEMMQAQTELYKALSSSSSDQNQNYKATHMIALEIGGGNGLQGMTLGASSNMDIPCV
FG06997.1 	LAGDELLEAQQELYKMCNTS---------ATHMISVEVGGANGLSGLLLGSSDQMDIPTV
AN7952.1  	LENDECLKAHEELMRATGSP--------QVSGFMALEIGGGNGV--LNLGVAARFGVFCI
          	  .*   .*  *: :  .          . : ::: *:**.**:  :  * :   .:  :

MG00722.1 	DGDLMG1RAYPNFEMVSQYVMG-NSIDELLPVTLCSGTGHNVVIPRGQPDAATASAAIRD
NCU00796.1	DGDWMG~RAYPTKWQTTPVVFN-ERSPIWSPIAVADGNGNVLVMPKAASDAAVEN-IIRA
FG06997.1 	DGDWMG~RAYPTKWQTTPVVFK-ERETIWSPIAVSDGNGNVLVMPKASSDEAVER-IIRS
AN7952.1  	DADYMG~RAYPTTWQVTANVYGTERGEALVPMTIASGDGSFITMTASRTDKLVDK-ILRA
          	*.* ** ****.   .:  *  ::      *:::..* *  :.:. . .*  .    :* 

MG00722.1 	KLIAMG2SAGGAAGNPITGAEMKHFGIPNTFSLAWRLGRAVVAAKSRPRAWSFADVARSI
NCU00796.1	ALSQMG~SQVGAADPPVTGAEMKRWVVENTVSQAWRLGRAVARARAMN---RLDEVAETI
FG06997.1 	ALSEMG~SQVGAADAPVTGAETRRWAIEHTLSQSWRIGRAVARARKEN---RVDNVAETI
AN7952.1  	SCVEMG~CRAGSAGPPKTSKTVREQAVTNTVSLAWWIGRAIALEKT------ISDRAKRM
          	    ** .  *:*. * *.   :.  : :*.* :* :***:.  :       . : *. :

MG00722.1 	IAECGGP---GCCRVVFRGKMRGVESRITTTAHSIGQVTIEKVDEEDDDDGLYD------
NCU00796.1	VEECGGGQKGAAAKMLFKGKIVGVR-RTLRMGHVYGECIIEGTDMTDDTTTTNHGRRTGG
FG06997.1 	IDECGGP---GAGKVLWKGKIIGVE-RTLRNGHVYGECLIEGADVRDEASAAS-------
AN7952.1  	IDELGGP---ESAAILGEGKITSVE-RVLRTGHTYGVLEVDGG-LPDGT-----------
          	: * **     .  :: .**: .*. *    .*  *   ::     *             

MG00722.1 	----------GEDAGSITEVKVPFMNENLSVIGRKADG-----------TEK0VLAMVPD
NCU00796.1	RRGGQGVDGEEKEDFIGKRIKIPFKNENIAAIRIPAEDGSNDENKELEKQED~VLAIVPD
FG06997.1 	--------GEVSEQQFKGIVKIPFKNENIAALKVHPD-------RKEELQED~VLAIVPD
AN7952.1  	----------------KVIIRIPFKNENAFVEAVLPTG-----------ESR~ILASVPD
          	                   :::** ***  .    . .            .  :** ***

MG00722.1 	LIMILDIYTGENVGVQDYRYGLKVAVMAMAPHPVWT-TKRGLEVGGPKAFGLP-YEYVPT
NCU00796.1	LISVIDAQSGEAVGTPEYRYGLLVIVIGIAASDKWTGSQRGIDLGGPKAFGFDHLKYEPL
FG06997.1 	LVCVIDAQNGEAVGTPEYRYGLLVVVLGIAASDRWTGSERGIKIGGPDAFGFEHLKFEPL
AN7952.1  	LIAVLDAETGAGLGTPEYKYGLKVMIIAIAASPRWTDTPRGMALGGPGSMGFDGIEYVPI
          	*: ::*  .*  :*. :*:*** * ::.:*.   **.: **: :*** ::*:   :: * 

MG00722.1 	LEYSKPRSVIDEFQSPCATAAP
NCU00796.1	GRFVKPVSVIDEFDRSIE----
FG06997.1 	GQYFKPRSVIDEYDEQ------
AN7952.1  	GKYRKPRSVIDAFAV-------
          	 .: ** **** :
```
